# Relationship between circadian rhythm and Malondialdehyde serum levels in acute and stabilized schizophrenic patients

**DOI:** 10.1192/j.eurpsy.2024.1580

**Published:** 2024-08-27

**Authors:** E. Díaz-Mesa, C. Cárdenes Moreno, A. Morera-Fumero, I. Perez-Sagaseta De Ilurdoz, P. Abreu-González, M. R. Cejas-Méndez, M. L. Fernández-López, M. S. Henry-Benítez

**Affiliations:** ^1^PSIQUIATRÍA, HOSPITAL UNIVERSITARIO DE CANARIAS; ^2^ MEDICINA INTERNA, PSIQUIATRÍA Y DERMATOLOGÍA; ^3^FISIOLOGÍA, UNIVERSIDAD DE LA LAGUNA, SAN CRISTOBAL DE LA LAGUNA, Spain

## Abstract

**Introduction:**

Malondialdehyde (MDA) is a product of polyunsaturated fatty acid peroxidation (Del Rio D, et al. A review of recent studies on MDA as toxic molecule and biological marker of oxidative stress. Nutr Metab Cardiovasc Dis. 2005;15:316-28). It is a biomarker of oxidative stress and is involved in the pathophysiology of schizophrenia (Goh et al. Asian J Psychiatr. 2022;67:102932). Schizofrenia is linked to disrupted oxidative balance and inflammation (Więdłocha et al. Brain Sci. 2023;13:490). Prior research has shown connections between biomarkers and circadian rhythms in schizophrenia (Morera & Abreu. Acta Physiol Scand. 2007;43:313-14) and diabetes type 2 (Kanabrocki EL, et al. Circadian variation in oxidative stress biomarkers in healthy and type II diabetic men. Chronobiol Int. 2002;19:423-39). To determinate if MDA levels have a role in schizophrenia and follow a circadian rhythm may be useful.

**Objectives:**

The aim of our study is to compare diurnal and nocturnal MDA serum levels in patients in acute and stabilized phases of schizophrenia according to CIE-10 to find out if there are variations related with circadian rhythms

**Methods:**

47 patients were included in our study in two clinical phases: acute episode and stabilization. Blood samples were collected at 12:00h and at 00:00h. MDA serum levels were measured twice: when patients were decompensated (admission) and at clinical stabilization (discharge). The relationship between quantitative variables at both times was analysed by T-Student test

**Results:**

There is no significative difference between night and day MDA levels in the acute phase of the schizophrenia (2.22±1.352 vs. 1.93±1.530, p<0.09). There is statistical significance between 12:00 and 00:00 (1.90±1.136 vs. 1.34±0.868, p<0.001) at discharge: it was observed that levels decreased. This result can be interpreted as there is circadian rhythm in stabilized phases.

**Conclusions:**

MDA levels in patients with schizophrenia do not follow a circadian rhythm in the acute episode. When they are clinically stabilized present a circadian change. These patients lose the circadian rhythm in acute episodes. MDA circadian rhythm may help diagnose the clinical phase and its severity. It is necessary to perform more studies to know its utility as an oxidative biomarker

**Disclosure of Interest:**

None Declared

